# Oxidative Stress in Kidney Diseases: The Cause or the Consequence?

**DOI:** 10.1007/s00005-017-0496-0

**Published:** 2017-12-06

**Authors:** Natalia Krata, Radosław Zagożdżon, Bartosz Foroncewicz, Krzysztof Mucha

**Affiliations:** 1 Department of Immunology, Transplantology and Internal Diseases, Medical University of Warsaw, Nowogrodzka 59, 02-006 Warsaw, Poland; 2 Institute of Biochemistry and Biophysics, Polish Academy of Sciences, Warsaw, Poland; 3 Department of Clinical Immunology, Medical University of Warsaw, Warsaw, Poland

**Keywords:** Oxidative stress, Biomarkers, Chronic kidney disease

## Abstract

Exaggerated oxidative stress (OS) is usually considered as a disturbance in regular function of an organism. The excessive levels of OS mediators may lead to major damage within the organism’s cells and tissues. Therefore, the OS-associated biomarkers may be considered as new diagnostic tools of various diseases. In nephrology, researchers are looking for alternative methods replacing the renal biopsy in patients with suspicion of chronic kidney disease (CKD). Currently, CKD is a frequent health problem in world population, which can lead to progressive loss of kidney function and eventually to end-stage renal disease. The course of CKD depends on the primary disease. It is assumed that one of the factors influencing the course of CKD might be OS. In the current work, we review whether monitoring the OS-associated biomarkers in nephrology patients can support the decision-making process regarding diagnosis, prognostication and treatment initiation.

## Introduction

**Fig. 1:**
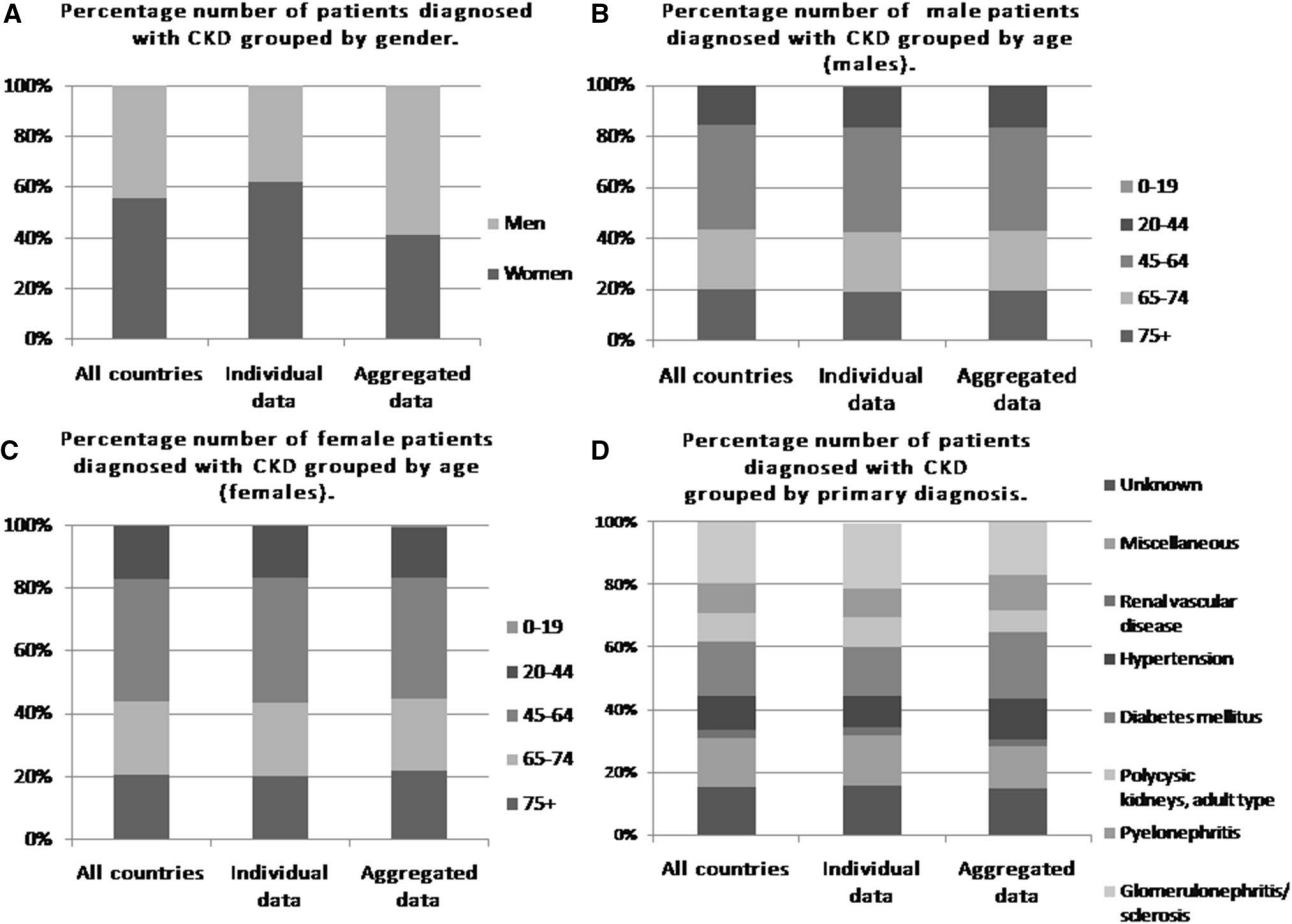
Percentage number of patients diagnosed with CKD grouped by various parameters such as: **a** gender, **b** age (males), **c** age (females), **d** primary diagnosis. Figures above summarize patients data presented in ERA-EDTA Registry annual report 2014 (Pippias et al. 2017)

Chronic kidney disease (CKD) is a public health problem that, depending on country, affects approximately 8–13% of population, involving both males and females (Fig. 1a) in all ages (Fig. 1b, c) (Bruck et al. 2016; Chronic Kidney Disease Prognosis et al. 2010). The main causes of CKD are: diabetes mellitus, hypertension, glomerulonephritis and cardiovascular diseases (Fig. 1d) (Mucha et al. 2016; Vassalotti et al. 2010). CKD is frequently progressive and the progression depends on both the primary disease as well as other factors, such as diet, smoking, coexisting obesity, etc. The initial suspicion of CKD is based on the clinical symptoms, such as proteinuria, erythrocyturia or hematuria, edema or hypertension. However, the final diagnosis must be confirmed by kidney biopsy (Mucha et al. 2016), which is an invasive diagnostic method—for that reason the researchers and nephrologists are looking for a safer and less-invasive diagnostic methods (Mucha et al. 2014). The researchers discovered numerous serum or urine components, that can contribute to the pathogenesis of kidney diseases and therefore, might be taken under consideration as potential biomarkers. However, there is still little consensus regarding the applicability of each of these markers and search for new biomarkers in CKD continues.

Some of the newly identified biomarkers in CKD are related to oxidative stress (OS). Indeed, it is currently known that several renal diseases might be related to coexisting OS (Small et al. 2012). Generally, exaggerated OS can be considered as a disturbance in regular function of organism’s cells and molecules. In order to control the OS level, the organism utilizes a natural ability to keep the balance between pro- and antioxidant systems (Scholze et al. 2016). It is believed that the main mediators of OS are reactive oxygen species (ROS). Transiently increased concentrations of ROS play a significant role in maintaining organism’s homeostasis, as they are a part of the redox-related signaling, and also in the immune defense system, as they are produced in high amounts in inflammation. However, the long-lasting excessive levels of ROS may lead to oxidation of DNA, lipids or proteins (Matsuyama et al. 2009; Small et al. 2012) and cause cellular damage in CKD patients (Scholze et al. 2016). Recently, this issue becomes even more important, as a number of redox modulators are considered as potential therapeutics in various human diseases, e.g., in a range of malignancies either as single compounds (Muchowicz et al. 2014; Trzeciecka et al. 2016) or in combinations with more classical therapies (Chou et al. 2017).

**Fig. 2:**
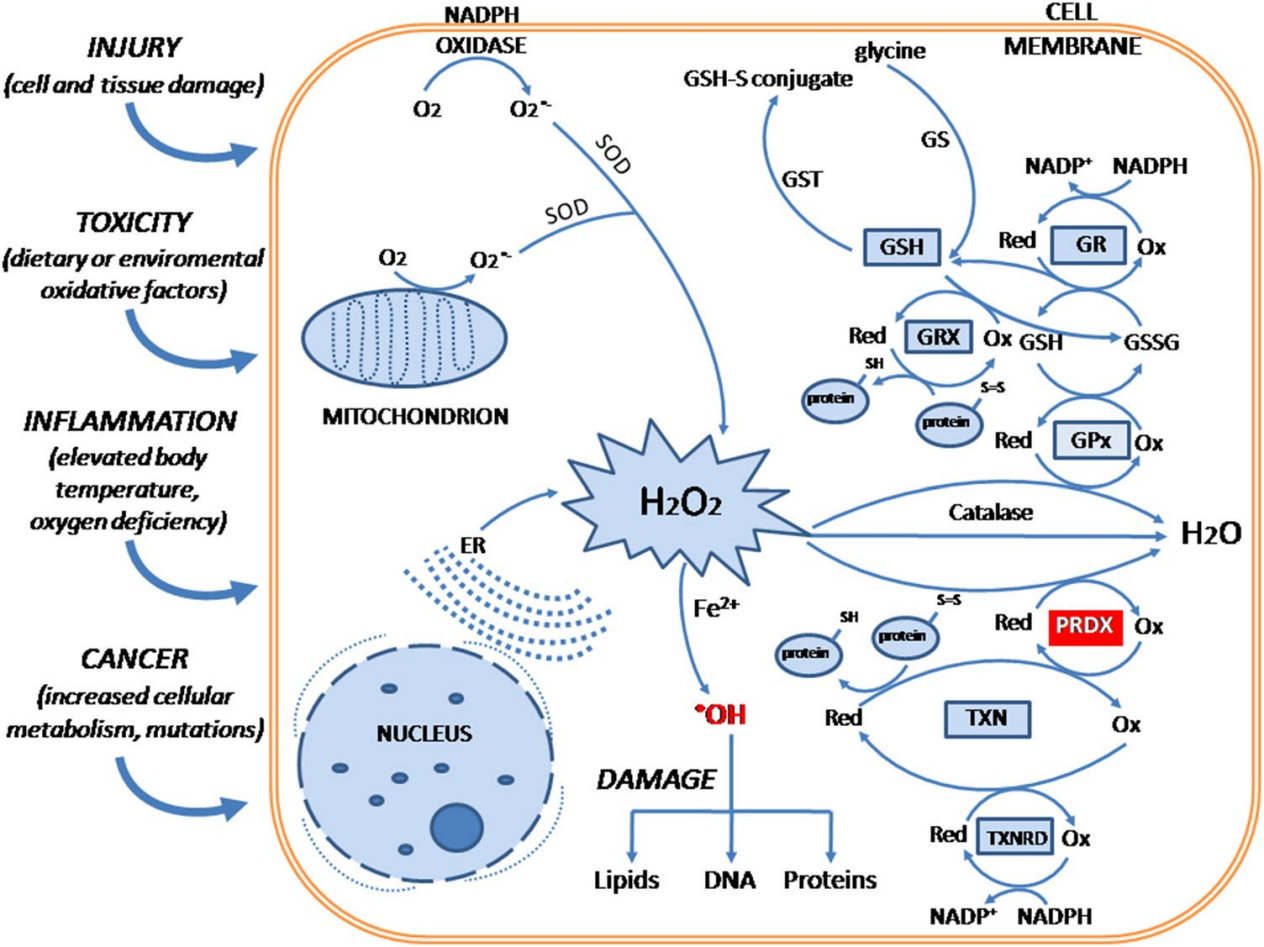
Cellular mechanisms related to oxidative stress. The mechanisms of superoxide anion radical (O_2_^·−^) scavenging by SOD—superoxide dismutase and formation of hydrogen peroxide (H_2_O_2_) and subsequent removal of toxic H_2_O_2_ by several antioxidant enzymes that have prognostic significance in various type of diseases. The antioxidants are marked as follows: *GSH* glutathione (reduced) and its oxidized form *GSSG* glutathione disulfide, *GR* glutathione reductase, *GRX* glutaredoxin, *GPx* glutathione peroxidase, *GST* glutathione *S*-transferase, *GS* glutamine synthetase, *PRDX* peroxiredoxin, *TXN* thioredoxin, *TXNRD* thioredoxin reductase, *NADPH* nicotinamide adenine dinucleotide phosphate oxidase

Endogenous ROS are generated by several main enzymatic processes such as cellular respiration (by the mitochondrial electron transport chain) or activity of nicotinamide adenine dinucleotide phosphate (NADPH) oxidase enzyme complex. Exogenous-derived ROS are usually induced by external factors, such as chemical pollutants, some drugs or UV light exposure. Among ROS, the most essential ones are free radicals: superoxide and hydroxyl (^·^HO), as well as non-radical molecules: hydrogen peroxide (H_2_O_2_) and singlet oxygen. On the other hand, ROS can be removed by our intrinsic enzymatic systems, such as superoxide dismutase (SOD), catalase, glutathione- and thiol-dependent enzymatic chains and other natural components (Fig. 2). There is also a range of antioxidant chemical agents that can be introduced to the organism, e.g., in a diet (Di Meo et al. 2016).

Regarding the kidney physiology, the main principle of proper redox regulation is to maintain the balance of electrolytes and physiological buffer systems to keep renal functions (Palm and Nordquist 2011). Additionally, kidneys remove a whole range of toxins and waste metabolites (Aveles et al. 2010), which otherwise would accumulate in the organism inducing an imbalance in redox homeostasis (Poulianiti et al. 2016). Therefore, OS should be both seen as a potential cause and a consequence of CKD.

## Biomarkers of OS

Potentially, a wide range of molecules can be used as OS biomarkers. All of the presented OS biomarkers can be measured, depending on their origin (e.g., serum, plasma, urine or tissue) or chemical form using various biotechnological techniques such as: enzyme-linked immunosorbent assays, radioimmunoassay, gas chromatography/mass spectrometry, liquid chromatography/mass spectrometry, immunolabeling, immunohistochemistry, etc. (Ho et al. 2013). Regardless of the disease, it ought to be mentioned that proper identification and validation of markers should follow a number of guidelines in order to produce meaningful observations (Brennan et al. 2010; O’Leary et al. 2014).

### Protein-Related Markers of OS

Among the cell constituents, the common target for ROS reactivity is the thiol side chain of a cysteine residue, but ROS can induce multiple other changes in the chemical structure of intracellular and extracellular proteins (Wang et al. 2012). Indeed, it has been observed that patients with chronic CKD have increased levels of plasma thiol oxidation and protein carbonylation that can indicate or even contribute to progressive renal dysfunction (Matsuyama et al. 2009). Other protein-related markers of OS include nitrotyrosine, myeloperoxidase (MPO) and oxidized low-density lipoprotein (OxLDL), as described below.

Nitrotyrosine (Tyr-NO_2_) is often considered as an oxidative/nitrosative stress biomarker in inflammatory diseases (Herce-Pagliai et al. 1998). Nitration of tyrosine can be used as an indicator of OS. Importantly, nitration of proteins and lipoproteins may also play a significant role in pathophysiology, e.g., the formation of foam cells which is mediated by macrophages that take up the nitrated LDL molecules (Graham et al. 1993).

The presence of MPO was detected in granules of human inflammatory cells such as macrophages, neutrophils and monocytes. MPO possesses the ability to convert H_2_O_2_ to various types of ROS including: ^·^OH, ONOO^−^, NO_2_ and HOCl which can then modify proteins, lipids or lipoproteins. Due to the ability to generate HOCl, a potent antimicrobial agent, the MPO plays a significant role in immune defense system. Elevated levels of circulating MPO have been found in patients diagnosed with cardiovascular disease (CVD) (Zhang et al. 2001). Prospective studies showed that high MPO levels predict increased risk of cardiovascular disease in healthy individuals (Meuwese et al. 2007). Likewise, in CKD patients altered functionality of MPO can indicate the risk of development of endothelial changes and cardiovascular complications (Kisic et al. 2016).

The accumulation of OxLDL stimulates production of pro-inflammatory cytokines by overlying endothelial cells, and therefore it becomes one of the main mediators of CVD development (Mertens and Holvoet 2001), also in patients with kidney diseases (Pandya et al. 2015). Interestingly, OxLDL has recently been reported to predict the development of renal dysfunction in atrial fibrillation (Polovina et al. 2016). However, in hemodialyzed patients OxLDL levels did not significantly correlate with the risk major adverse cardiac events (Wagner et al. 2017), so the applicability of this marker in renal disorders remains to be fully elucidated.

### Lipid-Related Markers of OS

Besides proteins, lipids are the extremely vulnerable substrates for oxidation because of their specific molecular structure and presence of reactive double bonds (Porter et al. 1995). The products of lipid oxidation that are considered as a biomarkers are: lipid hydroperoxides, fluorescent products of lipid peroxidation, oxidation resistance assays and oxysterols (Ho et al. 2013). Generally, human and animal research have shown that lipid oxidation plays a significant role in predicting the progression of cardiovascular and renal diseases and their response to therapies. In kidney diseases, there are reports of using such markers as: malondialdehyde (MDA), isoprostanes (IsoPs) or isolevuglandins (IsoLGs) (Scholze et al. 2016), as described below.

MDA is generated through peroxidation of polyunsaturated fatty acids; it interacts with proteins and is potentially atherogenic. MDA is typically investigated from plasma samples. The most popular method for MDA analysis is a colorimetric assay based on the reaction between MDA and thiobarbituric acid (TBA), also the TBA reacting substances (TBARS) assay are specific for MDA (Meagher and FitzGerald 2000). MDA determination and the TBA test can clearly explain the complex process of lipid peroxidation. By using the MDA analysis and/or the TBA test, also making a sample measurements interpretation, we may determine the OS within an organism (Trevisan et al. 2001). For instance, MDA measurements were carried out in a study that included a group of 38 children diagnosed with IgA nephropathy in order to assess whether the serum OS biomarkers levels measurements can be potentially used as a non-invasive diagnostic method in this disease (Pei et al. 2016). It was reported that the increased plasma level of advanced oxidation protein products and MDA were detected, and the SOD serum levels were decreased (Pei et al. 2016). Interestingly, it has been reported that serum MDA can be downregulated by a recently synthesized caffeic acid derivative: *N*-propyl caffeamide (Cheng et al. 2017), which belongs to a class of potent candidates for anti-inflammatory and cardioprotective drugs.

IsoPs are a family of stable, prostaglandin-like compounds. They are formed in vivo from the free radical-catalyzed peroxidation of essential fatty acids (primarily arachidonic acid) (Morrow et al. 1990, 1992). IsoPs are originally located on a cell membrane then they are released into circulation by phospholipases (Stafforini et al. 2006), and then accumulate in tissues, blood and urine. The most stable form of IsoPs that are considered as a potential biomarker are F_2_-IsoPs (with F-type prostane ring). The research concerning IsoPs levels in urine and plasma samples, collected from animals and humans, showed that there is a correlation between OS and levels of these biomarkers (Fam and Morrow 2003). IsoPs levels are also elevated in case of various OS-related risk factors such as diabetes mellitus, obesity, cigarette smoking, hypercholesterolemia, and hyperhomocysteinemia (Morrow 2005). Elevated F_2_-IsoPs level is also associated with renal vasoconstriction and major renal failure (Moore et al. 1998).

IsoLGs are products of cyclooxygenase and free radical-induced oxidation of arachidonates, they react rapidly and irreversibly with primary amines within the cell and form pyrrole (lactam) and oxidized pyrrole (hydroxylactam) adducts (Frijhoff et al. 2015). IsoLG adducts participate in pathological processes like induction of inflammation, immune response or cell death, and also act as inhibitors of ion channels. The results from research in animal models suggest that excessive levels of IsoLGs could be related to atherosclerosis, hypertension, inflammation, neurodegeneration or arrhythmia (Salomon and Bi 2015), but also renal disease (Degenhardt et al. 2002).

### Biomarkers of ROS-Induced DNA and RNA Oxidation

OS induces also oxidation of DNA and RNA, through the oxidation of nucleosides (particularly the guanine moiety), which are then excreted into the urine. The measurements of urine samples may be interpreted as an indicator of the cumulative body OS. Therefore, they are most suitable to conditions where OS affects all tissues in the body and less to elevated OS in minor organs. Although more than 100 oxidative DNA-modifications have been reported to date, the most frequently used is the 7,8-dihydro-8-oxo-2′-deoxyguanosine (8oxodG), which is one of the well-known OS biomarkers (Barregard et al. 2013), including kidney diseases (Schupp et al. 2016). While a large proportion of studies with this biomarker in renal diseases have been carried out in hemodialyzed patients, there are also indications that 8oxodG levels might correlate positively proteinuria in CKD patients (Nakamura et al. 2010). Importantly, 8oxodG can be also measured in urine, although the diagnostic significance of this method is not clear at present (Barregard et al. 2013).

It is worth mentioning that the deteriorating effects of OS on DNA structure modulate the activities of DNA-repair enzymes. Indeed, the polymorphisms of DNA-repair genes can affect the DNA-repair capacity and modulate patient’s susceptibility to renal disease. Such an effect has been reported in X-ray cross-complementing group 1 repair enzyme, where Arg399Gln polymorphism conferred the increased risk of development of end-stage renal disease (ESRD) (Trabulus et al. 2012). Also, measuring the presence and/or the activity of DNA-repair enzymes, such as 8-oxoguanine-DNA-*N*-glycosylase-1 (Cerrillos-Gutierrez et al. 2016), might help monitoring the altered OS conditions in renal patients.

### Biomarkers Related to Natural Antioxidant Capacity

While numerous works have evaluated the oxidant-related biomarkers in CKD, there is a scarcity of studies examining the natural body antioxidants in this disease. One of such studies has included systemic lupus erythematous (SLE) patients with nephritis (Lalwani et al. 2015). The results of this study have shown that decreased serum thiols levels are correlated to creatinine and serum C3 levels. Interestingly, it has been suggested that thiol levels may be used to differentiate between SLE patients with and without renal pathology (serum thiol levels are not affected by immunosuppressive drug therapy). It is possible that combination of thiols and creatinine or C3 serum levels may be used as a future predictive biomarker of chronicity of renal pathology in SLE patients (Lalwani et al. 2015).

## Oxidative Stress in a CKD Patient

### Causes

There are different mechanisms that could explain the existence of elevated OS in patients suffering from CKD. The basic characteristics of renal patients are as follows: advanced age, diabetes and renal hypertension, all of which predispose them to increasing levels of OS comparing to the general population. Another cause of OS in CKD patients is inflammation. There is a correlation between renal dysfunction and the mediators and markers of inflammation such as C-reactive protein, interleukin (IL)-6, tumor necrosis factor-α and fibrinogen, which proves that CKD is an inflammatory process itself (Cachofeiro et al. 2008). In response to activation of polymorphonuclear neutrophils, the MPO is generated and activated ROS excretion. Indeed, serum MPO was found to be associated with markers of inflammation in CKD patients (Kisic et al. 2016). The potential roles for other OS-related markers have been described above. Generally, the dysregulated systemic redox status can be already detected at an early stages of CKD (Miranda-Diaz et al. 2016) and it tends to increase along with the progression of the renal disease, which translates into gradual exaggeration of generalized tissue injury in CKD patients and contributes to CKD-associated comorbidities (Tucker et al. 2015). This exaggeration of OS mediators is not accompanied with an increase of antioxidant capacity of the body (Karamouzis et al. 2008), which induces a deepening imbalance in redox status of the CKD patient. Additionally, an obvious factor contributing to OS in late-stage CKD is a dysregulated metabolic waste disposal.

Notably, renal replacement therapy with maintenance hemodialysis does not improve the OS conditions in CKD patients after they progressed into ESRD. Conversely, each session of hemodialysis induces OS, because of ROS excretion on the surface of dialysis membranes (Peuchant et al. 1994), partly due to activation of phagocytes (Himmelfarb et al. 2001). Hemodialysis also tends to further exhaust the antioxidant capacity of the body (Jackson et al. 1995). Interestingly, vitamin E-coated dialysis membranes have been recently shown to reduce the levels of oxidative genetic damage in hemodialysis patients (Rodriguez-Ribera et al. 2017). Other studies suggested that applying cinacalcet (Ari et al. 2014) or lowering dialysate sodium (Macunluoglu et al. 2016) can improve systemic OS in maintenance hemodialysis patients. What is less understood are the causes underlying increased OS in patients undergoing continuous ambulatory peritoneal dialysis (CAPD) (Mehmetoglu et al. 2012). According to a recent study, in CAPD patients there is a noticeable distortion in OS management caused by increased enzymatic activity of SOD combined with a decrease in the activities of catalase and glutathione peroxidase (Ertan et al. 2017). Another study suggests that elevated plasma cyclophilin A can be one of the mediators of OS and inflammation in dialyzed patients (Jin and Vaziri 2017). Generally, however, patients undergoing CAPD are considered less predisposed to OS-related disorders than the hemodialyzed patients (Stepniewska et al. 2015).

### Consequences

Systemic OS can significantly contribute to endothelial dysfunction (Annuk et al. 2005) along with exaggeration of atherosclerosis (Esper et al. 2006) and development of CVD (Paoletti et al. 2005). As excessive ROS are genotoxic (Stopper et al. 2004; Stoyanova et al. 2010), OS may be a factor contributing to higher malignancy rates in ESRD patients (Shang et al. 2016). Also, the structural changes induced by ROS in β_2_-microglobulin are correlated with the incidence of amyloidosis due to inflammatory processes in renal patients (Capeillere-Blandin et al. 1991). Other OS-related problems in CKD include aggravation of hypertension (Mathis et al. 2012), and also neurologic disorders (oxidation of myelin), anemia (decrease in erythrocyte lifespan), inflammation (activation of nuclear factor κB: NF-κB), fibrosis and accelerated aging (reviewed in Vaziri 2004). Lastly, OS can alter multiple functions of the body through oxidation of hormones, such as parathyroid hormone (Hocher et al. 2012).

### Clinical Biomarkers

To help better understanding and monitoring of CKD progression there are few basic biomarkers such as serum creatinine, which is the main biomarker of kidney function used in clinical approach and it is used to estimate glomerular filtration rate (making eGFR), which correlates serum creatinine level with sex, age and weight of patient. However, those parameters are not fully applicable in diagnosis and prognosis of kidney injury. First of all, there are numerous limitations that enable using serum creatinine to estimate actual renal function, e.g., noticeable reduction of GFR can be present before a visible rise of creatinine level which may lead to irreversible loss of kidney function before the change of serum creatinine level. Moreover, creatinine in general is a poor biomarker that precludes the early diagnosis of acute renal injury and differentiation between various causes. That is why creatinine is an unreliable biomarker of kidney damage and there is a large need for searching an appropriate one (Malyszko 2010).

Recently, a new type of biomarker has been proposed, namely the neutrophil gelatinase-associated lipocalin (NGAL), the member of lipocalin family, which is expressed at low levels in several human tissues (including kidney) and possesses the ability to scavenge iron molecules, which are rapidly induced and released from the injured distal nephron (Mishra et al. 2003). Because of small molecular size (25 kDa) and resistance to degradation, NGAL is rapidly excreted and may be easily detected in urine. NGAL has recently been validated as a useful biomarker of CKD progression (Malyszko et al. 2008), and also an indicator of acute kidney injury in kidney transplantation patients (it may be used as a predictive biomarker for delayed graft function following kidney transplantation) (Malyszko et al. 2009). Importantly for this work, production of NGAL has been reported as an indicator of a response to OS before organ dysfunction can be detected by other biomarkers in acute kidney injury (Haase et al. 2011).

## Therapeutic Implications

### Control of the Disease Underlying CKD

Treatment of the underlying disease in CKD patients can directly translate into better control of OS conditions in their organs. This phenomenon is clearly noticeable, e.g., in diabetes patients, in which the proper control of glycemia can indeed alleviate OS and its consequences (Fiorentino et al. 2013). The same holds true for other diseases, such as autoimmune disorders or hypertension.

### Application of Antioxidants in CKD

The attempts of antioxidant therapy have been already conducted in randomized trials at various stages of renal disease, e.g., the study of Secondary Prevention with Antioxidants of Cardiovascular Disease in End-stage Renal Disease (SPACE) with the use of high-dose vitamin E. It was found that the hemodialyzed patients with a history of CVD, treated with high-dose vitamin E, had significantly reduced cardiovascular symptoms (Boaz et al. 2000). The positive results from the SPACE trial suggest that OS may be a major factor in cardiovascular disturbance in renal disease than in others. Also other research such as Prevention of Events with Angiotensin-Converting Enzyme Inhibition trial, foster the hypothesis about OS as particularly adverse in patients with CKD. It turned out that the angiotensin-converting enzyme inhibitor (trandolapril) reduce all-cause mortality only in patients with a GFR of 60 ml/min per 1.73 m^2^ comparing to those with a preserved renal function (Solomon et al. 2006). A study by Perna et al. (1991) has shown an impaired activity of alpha-ketoglutarate dehydrogenase of heart mitochondria in chronic renal failure. Further studies have led to conclusion that alpha-ketoglutarate (AKG), which is that one of the metabolites in Krebs cycle, possesses an ability to keep the redox balance within the cells. This molecule is involved in multiple metabolic and cellular pathways, e.g., serve as an energy donor, signaling molecule, regulator of epigenetic processes and precursor of amino acid biosynthesis. AKG also mediates in biosynthesis of collagen or regulation of gene expression (Zdzisinska et al. 2017). Indeed, some previous research (Long and Halliwell 2011; Sokolowska et al. 1999) suggests that AKG may be used as a therapeutic agent in states of protein deficiency and harmful OS conditions and could be used in kidney disease patients (Birck et al. 1999).

Despite these results, antioxidant therapies have not become a standard of care in renal patients up to date and more investigations are needed. It mainly remains unknown how antioxidant treatment can potentially alter the progression of CKD itself.

### Potential Antioxidant Agents in CKD

The future treatment therapies of CKD patients should be focused on reduction of ROS levels. One can suggest that it is already possible, e.g., with an application of several biological agents that may be used as an antioxidant treatment agents in CKD. According to Small et al. (2012) they are represented by group of organic compounds such as: vitamin A, C, E; beta carotene, *N*-acetyl cysteine (NAC) or flavonoids (e.g., resveratrol). Their main aim is to scavenge free radicals by incorporating into the cell plasma membrane, to block oxidation of lipids and DNA, which are the main causes of cellular damage. Vitamin E and C are usually delivered together, because in living organisms vitamin C participates in recycling of vitamin E, which increases antioxidant efficacy (Frei et al. 1990). NAC is considered an essential precursor to many endogenous antioxidant agents involved in the decomposition of ROS. l-cysteine is the limiting precursor to biosynthesis of glutathione. The sulfhydryl-thiol group of l-cysteine revealed also an ability to scavenge free radicals. The results of NAC supplementation in CKD patients have been variable. The NAC pretreatment showed the antioxidant properties by reducing ROS-dependent activity of NF-κB (Tumur et al. 2010), serum 8-isoprostane, MDA, and the inflammatory cytokine, e.g., IL-6 (Hsu et al. 2010; Nascimento et al. 2010). However, in general the results treatment of CKD patients with NAC were disappointing (Renke et al. 2010).

Other antioxidant agents that may be used in CKD therapy are omega-3 polyunsaturated fatty acids, represented by docosahexaenoic acid and eicosapentaenoic acid. They have been investigated in a large range of in vitro *and* in vivo CKD models. They support organisms’ antioxidant defense systems by enhancing γ-glutamyl-cysteinyl ligase and glutathione reductase levels (Arab et al. 2006). In progressive renal fibrosis models, the structure and function of kidneys were improved using docosahexaenoic and eicosapentaenoic acid supplementation. They also reduced inflammation, OS and tubulointerstitial fibrosis (Peake et al. 2011). Another antioxidant agent allopurinol and its metabolite, oxypurinol, are the inhibitors of xanthine oxidoreductase and possess the ability to decrease serum uric acid levels. Allopurinol treatment relies on blocking the reabsorption of uric acid (El-Sheikh et al. 2008) and has a protective effect in diseases involving OS. Several studies in human CKD patients show that allopurinol improved endothelial functions, prevented the increased left ventricular mass and slowed the progression of CKD by lowering patients serum uric acid levels (Kao et al. 2011; Siu et al. 2006).

The progression of CKD is strictly associated with atherosclerotic process. In regard to that, researchers proposed the use of a phenolic compound—resveratrol, which possess an antioxidant and anti-inflammatory properties. Through the modulation of mechanisms which are directly involved in OS and inflammation, resveratrol plays an important role controlling various metabolic disorders associated with CKD. It possesses ability to activate the transcription-related nuclear factor erythroid 2 or sirtulin-1 protein which are associated with the reduction of inflammation. These two agents are able to inhibit/antagonize the activity of the NF-κB, that participates in the inflammatory response (Saldanha et al. 2013). Although promising at the beginning, clinical studies have shown that there were no significant differences in pro-inflammatory or OS-related biomarkers in non-dialyzed CKD patients (Saldanha et al. 2016). However, additional studies with different times of treatment or doses should be conducted to better understand the effects of resveratrol supplementation.

In the grand majority of conducted trials, it was noticed that the effect of antioxidant therapy depends on the stage of CKD and for patients with advanced CKD stage 3 or 4, dialyzed or transplant recipients, there are some beneficial outcomes such as significantly reduced risk of ESRD and creatinine levels. In clinical research, antioxidant therapies require more time to confirm the applicability of various antioxidant agents as effective treatment methods.

### Potential Adverse Effects of Antioxidant Treatment

Several recent studies have suggested that excessive antioxidant treatment can be the cause of a range of adverse effects (Hamishehkar et al. 2016), including an increase in all-cause mortality (Miller et al. 2005), that especially holds true for an application of antioxidant vitamins in ESRD patients (reviewed in Kosmadakis et al. 2014). For instance, some studies suggest that supplementation with high doses of vitamin C may actually increase the lipid peroxidation (De Vriese et al. 2008) or may worsen clinical symptoms (Singer 2011) in hemodialysis patients. Therefore, caution and proper monitoring shall be advised for the use of antioxidants in ESRD patients until well-designed large clinical outcome trials are available.

## Conclusions

In summary, monitoring OS biomarker levels seems a promising way to improve nowadays diagnostic methods in CKD. The main question of the redox-focused studies in renal diseases is about the correlation between disturbance in balance of pro- and antioxidant systems and its influence for the development and progression of kidney failure. To study this phenomenon, it is needed to find a set of biomarkers that can be easily monitored and used for a non-invasive detection of redox disturbance in CKD. In future, it may help in the better understanding of the CKD etiology and make patient treatment and care more efficient.

## References

[CR1] Annuk M, Soveri I, Zilmer M (2005). Endothelial function, CRP and oxidative stress in chronic kidney disease. J Nephrol.

[CR2] Arab K, Rossary A, Flourie F (2006). Docosahexaenoic acid enhances the antioxidant response of human fibroblasts by upregulating gamma-glutamyl-cysteinyl ligase and glutathione reductase. Br J Nutr.

[CR3] Ari E, Kaya Y, Demir H (2014). Cinacalcet may improve oxidative DNA damage in maintenance hemodialysis patients: an observational study. Int Urol Nephrol.

[CR4] Aveles PR, Criminácio CR, Gonçalves S (2010). Association between biomarkers of carbonyl stress with increased systemic inflammatory response in different stages of chronic kidney disease and after renal transplantation. Nephron Clin Pract.

[CR5] Barregard L, Møller P, Henriksen T (2013). Human and methodological sources of variability in the measurement of urinary 8-oxo-7,8-dihydro-2′-deoxyguanosine. Antioxid Redox Signal.

[CR6] Birck R, Zimmermann E, Wassmer S (1999). Calcium ketoglutarate versus calcium acetate for treatment of hyperphosphataemia in patients on maintenance haemodialysis: a cross-over study. Nephrol Dial Transplant.

[CR7] Boaz M, Smetana S, Weinstein T (2000). Secondary Prevention with Antioxidants of Cardiovascular Disease in Endstage Renal Disease (SPACE): randomised placebo-controlled trial. Lancet.

[CR8] Brennan DJ, O’Connor DP, Rexhepaj E (2010). Antibody-based proteomics: fast-tracking molecular diagnostics in oncology. Nat Rev Cancer.

[CR9] Bruck K, Stel VS, Gambaro G (2016). CKD prevalence varies across the European general population. J Am Soc Nephrol.

[CR10] Cachofeiro V, Goicochea M, de Vinuesa SG (2008). Oxidative stress and inflammation, a link between chronic kidney disease and cardiovascular disease. Kidney Int Suppl.

[CR11] Capeillere-Blandin C, Delaveau T, Descamps-Latscha B (1991). Structural modifications of human beta 2 microglobulin treated with oxygen-derived radicals. Biochem J.

[CR12] Cerrillos-Gutierrez JI, Miranda-Díaz AG, Preciado-Rojas P (2016). The beneficial effects of renal transplantation on altered oxidative status of ESRD patients. Oxid Med Cell Longev.

[CR13] Cheng YY, Luo D, Xia Z (2017). In vivo cardioprotective effects and pharmacokinetic profile of *n*-propyl caffeamide against ischemia reperfusion injury. Arch Immunol Ther Exp.

[CR14] Chou HL, Fong Y, Wei CK (2017). A quinone-containing compound enhances camptothecin-induced apoptosis of lung cancer through modulating endogenous ROS and ERK signaling. Arch Immunol Ther Exp.

[CR15] Matsushita K, van der Velde M, Chronic Kidney Disease Prognosis Consortium (2010). Association of estimated glomerular filtration rate and albuminuria with all-cause and cardiovascular mortality in general population cohorts: a collaborative meta-analysis. Lancet.

[CR16] De Vriese AS, Borrey D, Mahieu E (2008). Oral vitamin C administration increases lipid peroxidation in hemodialysis patients. Nephron Clin Pract.

[CR17] Degenhardt TP, Alderson NL, Arrington DD (2002). Pyridoxamine inhibits early renal disease and dyslipidemia in the streptozotocin-diabetic rat. Kidney Int.

[CR18] Di Meo S, Reed TT, Venditti P (2016). Role of ROS and RNS sources in physiological and pathological conditions. Oxid Med Cell Longev.

[CR19] El-Sheikh AA, van den Heuvel JJ, Koenderink JB (2008). Effect of hypouricaemic and hyperuricaemic drugs on the renal urate efflux transporter, multidrug resistance protein 4. Br J Pharmacol.

[CR20] Ertan NZ, Bozfakioglu S, Ugurel E (2017). Alterations of erythrocyte rheology and cellular susceptibility in end stage renal disease: effects of peritoneal dialysis. PLoS One.

[CR21] Esper RJ, Nordaby RA, Vilarino JO (2006). Endothelial dysfunction: a comprehensive appraisal. Cardiovasc Diabetol.

[CR22] Fam SS, Morrow JD (2003). The isoprostanes: unique products of arachidonic acid oxidation—a review. Curr Med Chem.

[CR23] Fiorentino TV, Prioletta A, Zuo P (2013). Hyperglycemia-induced oxidative stress and its role in diabetes mellitus related cardiovascular diseases. Curr Pharm Des.

[CR24] Frei B, Kim MC, Ames BN (1990). Ubiquinol-10 is an effective lipid-soluble antioxidant at physiological concentrations. Proc Natl Acad Sci USA.

[CR25] Frijhoff J, Winyard PG, Zarkovic N (2015). Clinical relevance of biomarkers of oxidative stress. Antioxid Redox Signal.

[CR26] Graham A, Hogg N, Kalyanaraman B (1993). Peroxynitrite modification of low-density lipoprotein leads to recognition by the macrophage scavenger receptor. FEBS Lett.

[CR27] Haase M, Devarajan P, Haase-Fielitz A (2011). The outcome of neutrophil gelatinase-associated lipocalin-positive subclinical acute kidney injury: a multicenter pooled analysis of prospective studies. J Am Coll Cardiol.

[CR28] Hamishehkar H, Ranjdoost F, Asgharian P (2016). Vitamins, are they safe?. Adv Pharm Bull.

[CR29] Herce-Pagliai C, Kotecha S, Shuker DE (1998). Analytical methods for 3-nitrotyrosine as a marker of exposure to reactive nitrogen species: a review. Nitric Oxide.

[CR30] Himmelfarb J, McMenamin ME, Loseto G (2001). Myeloperoxidase-catalyzed 3-chlorotyrosine formation in dialysis patients. Free Radic Biol Med.

[CR31] Ho E, Karimi Galougahi K, Liu CC (2013). Biological markers of oxidative stress: applications to cardiovascular research and practice. Redox Biol.

[CR32] Hocher B, Armbruster FP, Stoeva S (2012). Measuring parathyroid hormone (PTH) in patients with oxidative stress—do we need a fourth generation parathyroid hormone assay?. PLoS One.

[CR33] Hsu SP, Chiang CK, Yang SY (2010). *N*-acetylcysteine for the management of anemia and oxidative stress in hemodialysis patients. Nephron Clin Pract.

[CR34] Jackson P, Loughrey CM, Lightbody JH (1995). Effect of hemodialysis on total antioxidant capacity and serum antioxidants in patients with chronic renal failure. Clin Chem.

[CR35] Jin K, Vaziri ND (2017). Elevated plasma cyclophillin A in hemodialysis and peritoneal dialysis patients: a novel link to systemic inflammation. Iran J Kidney Dis.

[CR36] Kao MP, Ang DS, Gandy SJ (2011). Allopurinol benefits left ventricular mass and endothelial dysfunction in chronic kidney disease. J Am Soc Nephrol.

[CR37] Karamouzis I, Sarafidis PA, Karamouzis M (2008). Increase in oxidative stress but not in antioxidant capacity with advancing stages of chronic kidney disease. Am J Nephrol.

[CR38] Kisic B, Miric D, Dragojevic I (2016). Role of myeloperoxidase in patients with chronic kidney disease. Oxid Med Cell Longev.

[CR39] Kosmadakis G, Da Costa Correia E, Carceles O (2014). Vitamins in dialysis: who, when and how much?. Ren Fail.

[CR40] Lalwani P, de Souza GK, de Lima DS (2015). Serum thiols as a biomarker of disease activity in lupus nephritis. PLoS One.

[CR41] Long LH, Halliwell B (2011). Artefacts in cell culture: alpha-ketoglutarate can scavenge hydrogen peroxide generated by ascorbate and epigallocatechin gallate in cell culture media. Biochem Biophys Res Commun.

[CR42] Macunluoglu B, Gumrukcuoglu HA, Atakan A (2016). Lowering dialysate sodium improves systemic oxidative stress in maintenance hemodialysis patients. Int Urol Nephrol.

[CR43] Malyszko J (2010). Biomarkers of acute kidney injury in different clinical settings: a time to change the paradigm?. Kidney Blood Press Res.

[CR44] Malyszko J, Bachorzewska-Gajewska H, Sitniewska E (2008). Serum neutrophil gelatinase-associated lipocalin as a marker of renal function in non-diabetic patients with stage 2–4 chronic kidney disease. Ren Fail.

[CR45] Malyszko J, Malyszko JS, Bachorzewska-Gajewska H (2009). Neutrophil gelatinase-associated lipocalin is a new and sensitive marker of kidney function in chronic kidney disease patients and renal allograft recipients. Transplant Proc.

[CR46] Mathis KW, Venegas-Pont M, Masterson CW (2012). Oxidative stress promotes hypertension and albuminuria during the autoimmune disease systemic lupus erythematosus. Hypertension.

[CR47] Matsuyama Y, Terawaki H, Terada T (2009). Albumin thiol oxidation and serum protein carbonyl formation are progressively enhanced with advancing stages of chronic kidney disease. Clin Exp Nephrol.

[CR48] Meagher EA, FitzGerald GA (2000). Indices of lipid peroxidation in vivo: strengths and limitations. Free Radic Biol Med.

[CR49] Mehmetoglu I, Yerlikaya FH, Kurban S (2012). Oxidative stress markers in hemodialysis and peritoneal dialysis patients, including coenzyme Q10 and ischemia-modified albumin. Int J Artif Organs.

[CR50] Mertens A, Holvoet P (2001). Oxidized LDL and HDL: antagonists in atherothrombosis. FASEB J.

[CR51] Meuwese MC, Stroes ES, Hazen SL (2007). Serum myeloperoxidase levels are associated with the future risk of coronary artery disease in apparently healthy individuals: the EPIC-Norfolk prospective population study. J Am Coll Cardiol.

[CR52] Miller ER, Pastor-Barriuso R, Dalal D (2005). Meta-analysis: high-dosage vitamin E supplementation may increase all-cause mortality. Ann Intern Med.

[CR53] Miranda-Diaz AG, Pazarin-Villasenor L, Yanowsky-Escatell FG (2016). Oxidative stress in diabetic nephropathy with early chronic kidney disease. J Diabetes Res.

[CR54] Mishra J, Ma Q, Prada A (2003). Identification of neutrophil gelatinase-associated lipocalin as a novel early urinary biomarker for ischemic renal injury. J Am Soc Nephrol.

[CR55] Moore KP, Holt SG, Patel RP (1998). A causative role for redox cycling of myoglobin and its inhibition by alkalinization in the pathogenesis and treatment of rhabdomyolysis-induced renal failure. J Biol Chem.

[CR56] Morrow JD (2005). Quantification of isoprostanes as indices of oxidant stress and the risk of atherosclerosis in humans. Arterioscler Thromb Vasc Biol.

[CR57] Morrow JD, Hill KE, Burk RF (1990). A series of prostaglandin F2-like compounds are produced in vivo in humans by a non-cyclooxygenase, free radical-catalyzed mechanism. Proc Natl Acad Sci USA.

[CR58] Morrow JD, Awad JA, Boss HJ (1992). Non-cyclooxygenase-derived prostanoids (F2-isoprostanes) are formed in situ on phospholipids. Proc Natl Acad Sci USA.

[CR59] Mucha K, Bakun M, Jaźwiec R (2014). Complement components, proteolysis related, and cell communication related proteins detected in urine proteomics are associated with IgA nephropathy. Pol Arch Med Wewn.

[CR60] Mucha K, Foroncewicz B, Paczek L (2016). How to diagnose and follow patients with glomerulonephritis without kidney biopsy?. Pol Arch Med Wewn.

[CR61] Muchowicz A, Firczuk M, Chlebowska J (2014). Adenanthin targets proteins involved in the regulation of disulphide bonds. Biochem Pharmacol.

[CR62] Nakamura T, Sato E, Fujiwara N (2010). Co-administration of ezetimibe enhances proteinuria-lowering effects of pitavastatin in chronic kidney disease patients partly via a cholesterol-independent manner. Pharmacol Res.

[CR63] Nascimento MM, Suliman ME, Silva M (2010). Effect of oral *N*-acetylcysteine treatment on plasma inflammatory and oxidative stress markers in peritoneal dialysis patients: a placebo-controlled study. Perit Dial Int.

[CR64] O’Leary PC, Terrile M, Bajor M (2014). Peroxiredoxin-1 protects estrogen receptor alpha from oxidative stress-induced suppression and is a protein biomarker of favorable prognosis in breast cancer. Breast Cancer Res.

[CR65] Palm F, Nordquist L (2011). Renal oxidative stress, oxygenation, and hypertension. Am J Physiol Regul Integr Comp Physiol.

[CR66] Pandya V, Rao A, Chaudhary K (2015). Lipid abnormalities in kidney disease and management strategies. World J Nephrol.

[CR67] Paoletti E, Bellino D, Cassottana P (2005). Left ventricular hypertrophy in nondiabetic predialysis CKD. Am J Kidney Dis.

[CR68] Peake JM, Gobe GC, Fassett RG (2011). The effects of dietary fish oil on inflammation, fibrosis and oxidative stress associated with obstructive renal injury in rats. Mol Nutr Food Res.

[CR69] Pei Y, Xu Y, Ruan J (2016). Plasma oxidative stress level of IgA nephropathy in children and the effect of early intervention with angiotensin-converting enzyme inhibitors. J Renin Angiotensin Aldosterone Syst.

[CR70] Perna AF, Zayed MA, Massry SG (1991). Impaired activity of alpha-ketoglutarate dehydrogenase of heart mitochondria in chronic renal failure: role of secondary hyperparathyroidism. Nephron.

[CR71] Peuchant E, Carbonneau MA, Dubourg L (1994). Lipoperoxidation in plasma and red blood cells of patients undergoing haemodialysis: vitamins A, E, and iron status. Free Radic Biol Med.

[CR72] Pippias M, Kramer A, Noordzij M (2017). The European Renal Association—European Dialysis and Transplant Association Registry annual report 2014: a summary. Clin Kidney J.

[CR73] Polovina M, Petrovic I, Brkovic V (2016). Oxidized low-density lipoprotein predicts the development of renal dysfunction in atrial fibrillation. Cardioren Med.

[CR74] Porter NA, Caldwell SE, Mills KA (1995). Mechanisms of free radical oxidation of unsaturated lipids. Lipids.

[CR75] Poulianiti KP, Kaltsatou A, Mitrou GI (2016). Systemic redox imbalance in chronic kidney disease: a systematic review. Oxid Med Cell Longev.

[CR76] Renke M, Tylicki L, Rutkowski P (2010). The effect of *N*-acetylcysteine on blood pressure and markers of cardiovascular risk in non-diabetic patients with chronic kidney disease: a placebo-controlled, randomized, cross-over study. Med Sci Monit.

[CR77] Rodriguez-Ribera L, Corredor Z, Silva I (2017). Vitamin E-coated dialysis membranes reduce the levels of oxidative genetic damage in hemodialysis patients. Mutat Res.

[CR78] Saldanha JF, Leal Vde O, Stenvinkel P et al (2013) Resveratrol: why is it a promising therapy for chronic kidney disease patients?. Oxid Med Cell Longev 2013:963217 10.1155/2013/963217 PMC3893857 24489988

[CR79] Saldanha JF, Leal VO, Rizzetto F (2016). Effects of resveratrol supplementation in Nrf2 and NF-kappaB expressions in nondialyzed chronic kidney disease patients: a randomized, double-blind, placebo-controlled, crossover clinical trial. J Ren Nutr.

[CR80] Salomon RG, Bi W (2015). Isolevuglandin adducts in disease. Antioxid Redox Signal.

[CR81] Scholze A, Jankowski J, Pedraza-Chaverri J (2016). Oxidative stress in chronic kidney disease. Oxid Med Cell Longev.

[CR82] Schupp N, Stopper H, Heidland A (2016). DNA damage in chronic kidney disease: evaluation of clinical biomarkers. Oxid Med Cell Longev.

[CR83] Shang W, Huang L, Li L (2016). Cancer risk in patients receiving renal replacement therapy: a meta-analysis of cohort studies. Mol Clin Oncol.

[CR84] Singer RF (2011). Vitamin C supplementation in kidney failure: effect on uraemic symptoms. Nephrol Dial Transplant.

[CR85] Siu YP, Leung KT, Tong MK (2006). Use of allopurinol in slowing the progression of renal disease through its ability to lower serum uric acid level. Am J Kidney Dis.

[CR86] Small DM, Coombes JS, Bennett N (2012). Oxidative stress, anti-oxidant therapies and chronic kidney disease. Nephrology.

[CR87] Sokolowska M, Oleszek A, Wlodek L (1999). Protective effect of alpha-keto acids on the oxidative hemolysis. Pol J Pharmacol.

[CR88] Solomon SD, Rice MM, Jablonski A (2006). Renal function and effectiveness of angiotensin-converting enzyme inhibitor therapy in patients with chronic stable coronary disease in the Prevention of Events with ACE inhibition (PEACE) trial. Circulation.

[CR89] Stafforini DM, Sheller JR, Blackwell TS (2006). Release of free F2-isoprostanes from esterified phospholipids is catalyzed by intracellular and plasma platelet-activating factor acetylhydrolases. J Biol Chem.

[CR90] Stepniewska J, Golembiewska E, Dolegowska B (2015). Oxidative stress and antioxidative enzyme activities in chronic kidney disease and different types of renal replacement therapy. Curr Protein Pept Sci.

[CR91] Stopper H, Schupp N, Bahner U (2004). Genomic damage in end-stage renal failure: potential involvement of advanced glycation end products and carbonyl stress. Semin Nephrol.

[CR92] Stoyanova E, Sandoval SB, Zúñiga LA (2010). Oxidative DNA damage in chronic renal failure patients. Nephrol Dial Transplant.

[CR93] Trabulus S, Guven GS, Altiparmak MR (2012). DNA repair XRCC1 Arg399Gln polymorphism is associated with the risk of development of end-stage renal disease. Mol Biol Rep.

[CR94] Trevisan M, Browne R, Ram M (2001). Correlates of markers of oxidative status in the general population. Am J Epidemiol.

[CR95] Trzeciecka A, Klossowski S, Bajor M (2016). Dimeric peroxiredoxins are druggable targets in human Burkitt lymphoma. Oncotarget.

[CR96] Tucker PS, Scanlan AT, Dalbo VJ (2015). Chronic kidney disease influences multiple systems: describing the relationship between oxidative stress, inflammation, kidney damage, and concomitant disease. Oxid Med Cell Longev.

[CR97] Tumur Z, Shimizu H, Enomoto A (2010). Indoxyl sulfate upregulates expression of ICAM-1 and MCP-1 by oxidative stress-induced NF-kappaB activation. Am J Nephrol.

[CR98] Vassalotti JA, Fox CH, Becker BN (2010). Risk factors and screening for chronic kidney disease. Adv Chronic Kidney Dis.

[CR99] Vaziri ND (2004). Oxidative stress in uremia: nature, mechanisms, and potential consequences. Semin Nephrol.

[CR100] Wagner S, Apetrii M, Massy ZA (2017). Oxidized LDL, statin use, morbidity, and mortality in patients receiving maintenance hemodialysis. Free Radic Res.

[CR101] Wang Y, Yang J, Yi J (2012). Redox sensing by proteins: oxidative modifications on cysteines and the consequent events. Antioxid Redox Signal.

[CR102] Zdzisinska B, Zurek A, Kandefer-Szerszen M (2017). Alpha-ketoglutarate as a molecule with pleiotropic activity: well-known and novel possibilities of therapeutic use. Arch Immunol Ther Exp.

[CR103] Zhang R, Brennan ML, Fu X (2001). Association between myeloperoxidase levels and risk of coronary artery disease. JAMA.

